# Laparoscopic anterior versus endoscopic posterior approach for adrenalectomy: a shift to a new golden standard?

**DOI:** 10.1007/s00423-016-1533-x

**Published:** 2016-11-26

**Authors:** O. M. Vrielink, K. P. Wevers, J. W. Kist, I. H. M. Borel Rinkes, P. H. J. Hemmer, M. R. Vriens, J. de Vries, S. Kruijff

**Affiliations:** 1 Department of Surgical Oncology, University Medical Center Groningen, P.O. Box 30.001, 9700 RB Groningen, the Netherlands; 2 Department of Surgical Oncology, University Medical Center Utrecht, Utrecht, the Netherlands

**Keywords:** Adrenalectomy, Transabdominal, Retroperitoneal, Approach, Laparoscopy

## Abstract

**Purpose:**

There has been an increased utilization of the posterior retroperitoneal approach (PRA) for adrenalectomy alongside the “classic” laparoscopic transabdominal technique (LTA). The aim of this study was to compare both procedures based on outcome variables at various ranges of tumor size.

**Methods:**

A retrospective analysis was performed on 204 laparoscopic transabdominal (UMC Groningen) and 57 retroperitoneal (UMC Utrecht) adrenalectomies between 1998 and 2013. We applied a univariate and multivariate regression analysis. Mann-Whitney and chi-squared tests were used to compare outcome variables between both approaches.

**Results:**

Both mean operation time and median blood loss were significantly lower in the PRA group with 102.1 (SD 33.5) vs. 173.3 (SD 59.1) minutes (*p* < 0.001) and 0 (0–200) vs. 50 (0–1000) milliliters (*p* < 0.001), respectively. The shorter operation time in PRA was independent of tumor size. Complication rates were higher in the LTA (19.1%) compared to PRA (8.8%). There was no significant difference in recovery time between both approaches.

**Conclusions:**

Application of the PRA decreases operation time, blood loss, and complication rates compared to LTA. This might encourage institutions that use the LTA to start using PRA in patients with adrenal tumors, independent of tumor size.

## Introduction

Since the first laparoscopic adrenalectomy in 1992 by Gagner et al. [1] and Higashihare et al. [2], many studies have shown the safety and feasibility of this technique [3–5]. In the last two decades, this procedure has grown to replace most open surgeries due to its various advantages, including decreased postoperative pain, shorter hospital stay, fewer complications, and decreased blood loss [6–8]. Especially for benign and small**-** to medium**-**sized adrenal tumors, laparoscopic adrenalectomy became the gold standard surgical procedure [9]. Conventional open adrenalectomy was still preferred for large tumors and malignancies [10]. However, as the technique and instruments improved, laparoscopic adrenalectomy seemed to be feasible for larger (>6 cm) and potentially malignant tumors [11]. For adrenocortical carcinoma, open surgery is still preferred to assure radicality (R0 resection) and to reduce the risk of tumor spill and the associated chance of local recurrence [12, 13].

Nowadays, different endoscopic approaches exist for adrenalectomy, whereof the laparoscopic transperitoneal adrenalectomy (LTA) is the most frequently performed [11]. The posterior location of the adrenal glands in the upper retroperitoneal space has influenced surgeons to move to the retroperitoneal approach. This approach, the posterior retroperitoneal adrenalectomy (PRA), standardized by Walz et al. [14], offers a more direct route to the adrenal gland without the need for mobilizing fragile organs such as the liver, pancreas, and spleen potentially saving time, surgical trauma, and complications.

Despite convincing data, many surgeons still prefer the LTA because of the familiar operative field and wider working space. Well-designed studies have been performed comparing both procedures, showing that outcomes of PRA were superior to LTA in terms of shorter operation time, less blood loss, and lower postoperative pain [15–17]. However, the adrenalectomies in those studies were performed on selected patients with small- to medium-sized adrenal tumors.

The University Medical Center Groningen (UMCG) has developed a two-decade experience performing LTA whereas the University Medical Center Utrecht (UMCU) has developed a significant experience in the PRA. The aim of this study was to compare both surgical procedures (LTA versus PRA) in patients with unilateral adrenal tumors at various ranges of tumor size performed by two respected academic centers in the Netherlands.

## Material and methods

### Study design and participants

A multicenter retrospective study was performed at the Department of Surgical Oncology of the University Medical Center Groningen (UMCG) and University Medical Center Utrecht (UMCU). For this type of study, formal consent is not required.

All patients with a unilateral adrenal tumor who underwent adrenalectomy using LTA between April 1998 and August 2013 at the UMCG where included. Furthermore, all patients with a unilateral adrenal tumor who underwent adrenalectomy using PRA between January 2008 and October 2013 at the UMCU were included. During this period, each center only performed the investigated surgical approach for adrenalectomy (LTA at UMCG and PRA at UMCU).

Exclusion criteria were previous major abdominal surgery, previous adrenal surgery, or open surgery in the medical history. Baseline demographic information, including patient’s age, gender, previous medical history, and body mass index (BMI) were obtained from digital patient files stored in the electronic databases of both university medical centers.

### Primary and secondary endpoints

The primary outcome measure was length of surgery (minutes) between initial incision and closure of the surgical wound. Secondary outcome measures were recovery time (days) and blood loss during surgery (in ml). The recovery time was defined as the time from surgery until discharge from clinic (in days).

The tumor size used for comparison is the largest diameter of the tumor recorded from the pathology results (in cm). Tumor sizes were categorized as small (≤ 4 cm), medium (>4 and ≤8 cm), and large (>8 cm). Lastly, both intra- and postoperative complications were scored as well as the conversion rate. Complications were classified according to the Clavien-Dindo classification [18].

### Surgical procedures

LTA was performed with the patient in the lateral decubitus position with the affected side facing upward. A total of three ports were placed in the subcostal area. For right adrenalectomy, one additional port was required for retraction of the liver. After CO2 insufflation (10 mmHg), the spleen, pancreas, and splenic flexure of the colon were detached from the retroperitoneum for left adrenalectomy and the triangular ligament of the liver was dissected and rotated medially for right adrenalectomy. The adrenal gland was exposed after dissection. Adrenalectomy was performed by ligation of the adrenal vein. The resected adrenal gland was placed in an endobag and pulled out through a port site.

**Fig. 1:**
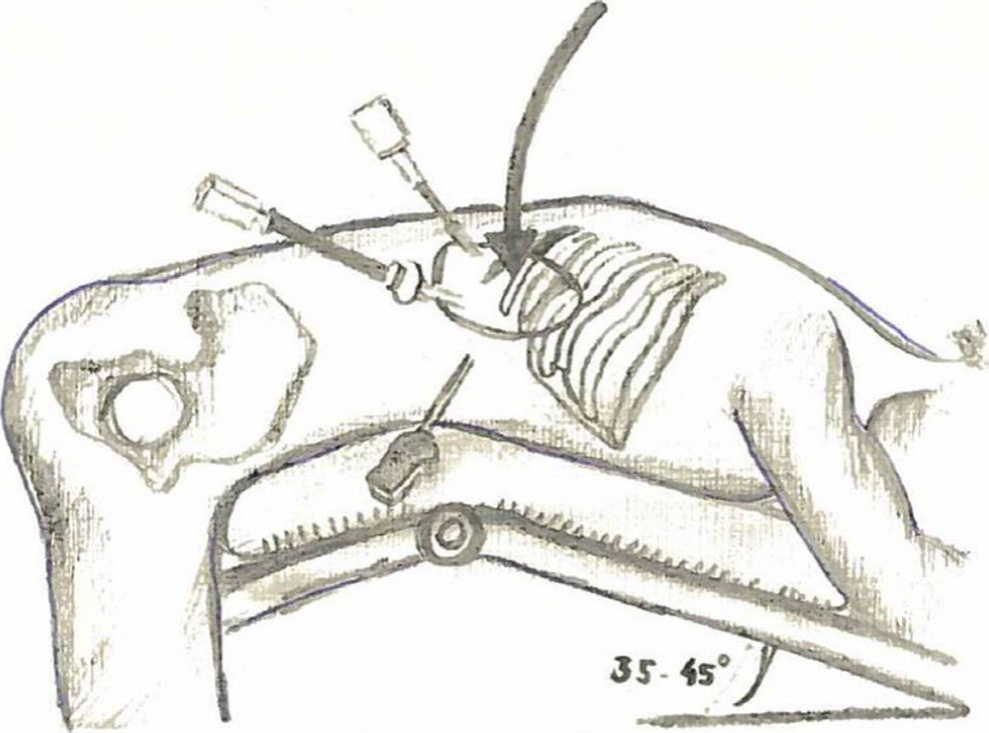
Schematic positioning of a patient in the prone position during PRA

**Fig. 2:**
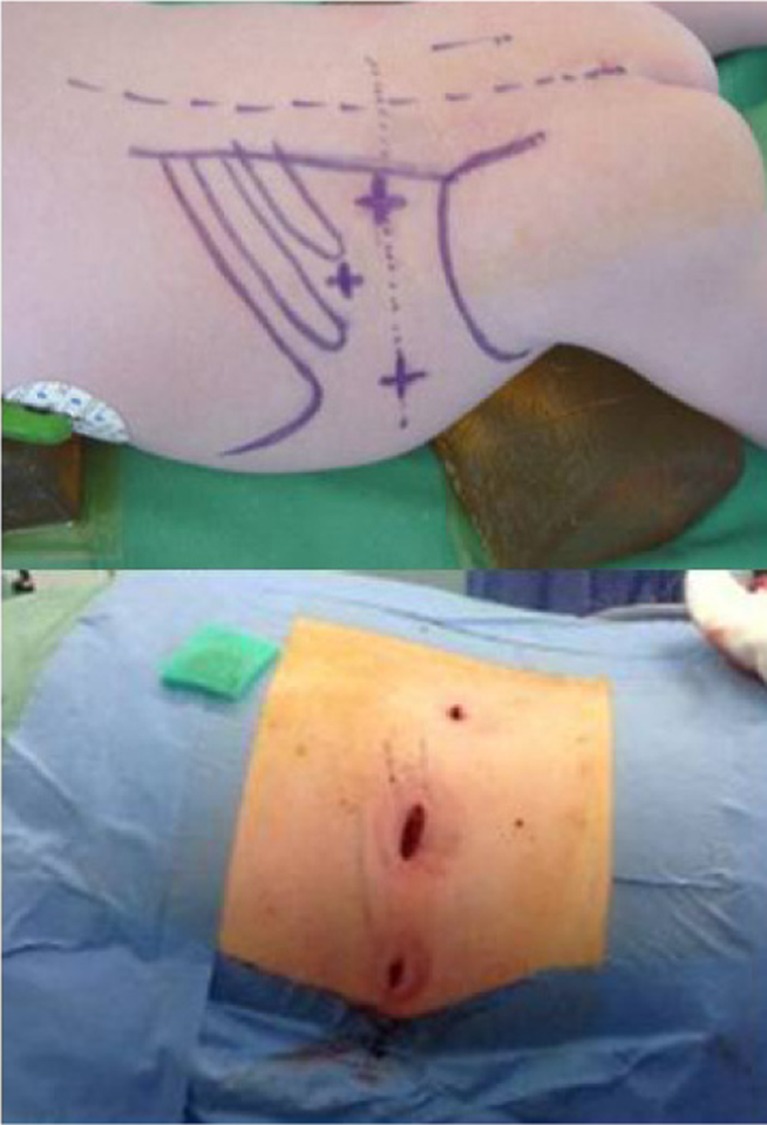
Incision points during PRA

PRA was performed with the patient in the prone position (Fig. 1). The first incision was made just below the tip of the 12th rib, and the retroperitoneal space was bluntly dissected with a finger. The second and third ports were then placed blind on the finger (Fig. 2). After CO2 insufflation (15–20 mmHg), fatty tissue from the posterior aspect of the kidney was dissected and the superior pole of the kidney was exposed. Adrenalectomy was performed by detaching the adrenal gland from adjacent structures and ligation of the adrenal vein. The resected adrenal gland was placed in an endobag and pulled out through the first incision site.

All surgical procedures at the UMCU were performed by one experienced laparoscopic surgeon and in the UMCG more than 93% of operations were performed or supervised by the same surgeon. Pre- and postoperative patients were, if necessary, monitored by the endocrinologist (according to hospital protocols) for the prevention of hypoaldosteronism.

### Statistical analyses

SPSS Statistics, version 22 was used for statistical analyses. Continuous variables were described as mean ± standard deviation (SD) or median with interquartile range (IQR). Categorical variables were described as count (*n*) and percentage (%).

Baseline patient characteristics of both groups were compared using the independent sample *t* test or the Mann-Whitney *U* test for continuous variables and the Pearson chi-square test for nominal variables. A *p* value <0.05 was considered statistically significant. Correlation analyses were performed using Pearson’s *r* or Spearman’s rank correlation to investigate the relationship between the outcome measures and explanatory variables. Multivariate linear regression analysis was performed to adjust for possible confounders with the inclusion of all possible variables with a significance level of *p* < 0.20 in univariate analyses. For the LTA procedures, a sub analysis was performed to compare outcome between procedures supervised in a teaching setting versus procedures that were performed by the experienced surgeon. Also, the influence of the year of the procedure was explored.

## Results

### Patient characteristics

Between April 1998 and August 2013, a total of 273 adrenalectomies were performed in the UMCG and UMCU. After applying the aforementioned inclusion and exclusion criteria, a total of 261 patients were included, whereof 204 LTA (UMCG) and 57 PRA (UMCU) procedures. PRA procedures were all performed between 2008 and 2013. Patient characteristics are listed in Table 1. The mean age of all patients was 51.1 (SD 15.0) years, consisting of 158 females (60.5%) and 103 (39.5%) males. In the LTA group, the median BMI was 26.4 (12.5–60.8) and in the PRA group, 25.8 (18.9–54.5). The median adrenal tumor size of all patients was 5.5 cm (range 0.50–15.0). The tumors were classified as 58 (23.6%) small, 160 (64.0%) medium, and 28 (11.4%) large tumors. There were no significant differences in baseline characteristics between both groups (Table 1).

**Table 1:** Baseline patient characteristics

	LTA (*n* = 204)	PRA (*n* = 57)	*p* value
Age	50.5 (SD 15.4)	53.5 (SD 13.3)	0.189*
Gender			0.644**
Female	125 (61.3%)	33 (57.9%)	
Male	79 (38.7%)	24 (42.1%)	
BMI, kg/m^2^	26.4 (12.5–60.8)	25.8 (18.9–54.5)	0.956***
Size, cm	5.7 (0.50–15.0)	5.5 (1.20–10.0)	0.838***
Size			0.729**
Small	44 (23.3%)	14 (24.6%)	
Medium	125 (66.1%)	35 (61.4%)	
Large	20 (10.6%)	8 (14.0%)	

*BMI* body mass index

*Independent samples *t* test, **Chi^2^ test, ***Mann-Whitney *U* test

### Factors associated with operation time, recovery time, and blood loss

Univariate analysis revealed surgical approach (*p* < 0.001) and tumor size (*p* < 0.001) to be associated with operation time. In LTA, the mean operation time was 173.3 (SD 59.1) minutes versus 102.1 (SD 33.5) minutes in PRA. Mean operation time was 141.7 (SD 46.1) minutes in small, 154.2 (SD 56.2) minutes in medium, and 183.6 (SD 90.7) minutes in large adrenal masses.

None of the variables correlated significantly with recovery time. Median recovery time in LTA was 4 (1–33) days versus 3 (1–20) days in PRA. Surgical approach was significantly associated with blood loss (*p* < 0.001). Median blood loss in LTA was 50 (0–1000) milliliters versus 0 (0–200) milliliters in PRA.

Correspondingly, the covariates age and BMI showed no significant relationship with any of the outcome variables (Table 2).

**Table 2:** Predictor variables and their association with the outcome variables

	*N* (%)	Operation time (min)	*p* value	Recovery time (days)	*p* value	Blood loss (ml)	*p* value
Approach			<0.001*		0.092**		<0.001**
LTA	204 (78.2)	173.3 (59.1)		4 (1–33)		50 (0–1000)	
PRA	57 (21.8)	102.1 (33.5)		3 (1–20)		0 (0–200)	
Gender			0.109**		0.080**		0.211**
Female	158 (60.5)	155.2 (67.8)		4 (1–33)		30 (0–1000)	
Male	103 (39.4)	161.3 (51.8)		3 (1–28)		35 (0–750)	
Age	51.1 (SD 15.0)		0.772***		0.536****		0.403****
<50	118 (45.4)	155.5 (64.2)		4 (1–21)		50 (0–750)	
≥50	142 (54.6)	160.2 (59.8)		3 (1–33)		25 (0–1000)	
BMI, kg/m^2^	26.2 (12.5–60.8)		0.600***		0.733****		0.646****
0–20	15 (7.6)	184.3 (54.5)		5 (2–33)		200 (0–400)	
≥20–25	59 (30.0)	140.0 (78.9)		3 (1–21)		0 (0–500)	
≥25–30	77 (39.1)	154.2 (58.5)		3 (1–25)		10 (0–1000)	
≥30	46 (23.4)	162.3 (52.8)		4 (1–19)		0 (0–500)	
Tumor size	5.5 (0.50–21.0)		<0.001***		0.119****		0.743****
Small	58 (23.6)	141.7 (46.1)		3 (1–21)		0 (0–500)	
Medium	160 (65.0)	154.2 (56.2)		3 (1–33)		30 (0–750)	
Large	28 (11.4)	183.6 (90.7)		4 (2–20)		25 (0–400)	

*BMI* body mass index

Level of significance *p* < 0.05 rendered in italics

*Independent samples *t* test, **Mann-Whitney *U* test, ***Pearson, ****Spearman

Subsequently, a multivariate analysis was performed analyzing the primary outcome measure, operation time. In this analysis surgical approach, tumor size, age, and gender were entered as independent variables because of *p* < 0.20 in univariate analysis. The multivariate analysis showed that surgical approach (*p* < 0.001) and tumor size (*p* < 0.001) were independent explanatory variables for operation time (Table 3).

**Table 3:** Multivariate analysis of explanatory variables for operation time

	Operation time		
	B	95% CI	*p* value
Approach	−69.0	−84.07–−53.98	*<0.001*
Tumor size	6.69	4.04–9.34	*<0.001*
Gender	8.66	−4.28–21.60	0.189
Age	1.23	−11.52–13.97	0.850

*B* beta, *CI* confidence interval

Level of significance *p* < 0.05 rendered in italics

For the LTA, a subgroup analysis was performed to compare the outcome of operations performed by the experienced surgeon versus operations supervised in a teaching setting by the same experienced surgeon at the UMCG. No significant difference in operation time (*p* = 0.663), recovery time (*p* = 0.711), blood loss (*p* = 0.415), and complications (*p* = 0.854) were found.

### Operation time

Table 4 shows the mean operation time for different tumor size categories per surgical approach. In PRA, the operation time is significantly shorter than in LTA for all tumor size categories. The operation time was the longest in large (>8 cm) adrenal masses with 205.3 (SD 98.3) minutes in LTA and 129.4 (SD 28.5) minutes in PRA.

**Table 4:** Operation time for different tumor sizes per surgical approach

	LTA mean (SD)	PRA mean (SD)	*p* value*	LTA (2008–2013) mean (SD)	*p* value*
Tumor size					
Small	156.5 (40.6)	95.0 (27.6)	*<0.001*	168.9 (39.3)	*<0.001*
Medium	169.8 (51.0)	98.7 (34.4)	*<0.001*	174.5 (37.4)	*<0.001*
Large	205.3 (98.3)	129.4 (28.5)	*0.001*	215.4 (106.6)	*0.037*

Level of significance *p* < 0.05 rendered in italics

*Independent samples *t* test

There was no significant difference in operation time in patients treated by LTA between 1998 and 2007 and patients treated by LTA between 2008 and 2013 (*p* = 0.092). Operation time in PRA was significantly shorter than operation time in LTA in patients treated between 2008 and 2013, independent of tumor size (Table 4).

Comparing LTA procedures performed in 1998–2007 with those in 2008–2013, no significant difference was found in recovery time (*p* = 0.160) and complications (*p* = 0.369). However, we did find a significant difference in blood loss (*p* = 0.001), with more blood loss in LTA between 1998 and 2007.

### Complications

Complication rates were higher in LTA (39; 19.1%) compared to PRA (5; 8.8%) (Table 5). There were ten conversions to open surgery in the LTA versus none in the PRA. Most complications were grade 2 complications. Hypokalemia and hypotension occurred most frequently (grade 2).

**Table 5:** Complication and conversion rates

	LTA no. (%)	PRA no. (%)	*p* value*
Complication			0.212
Grade 1	4 (1.96)	1 (1.75)	
Grade 2	21 (10.29)	4 (7.02)	
Grade 3	3 (1.47)	0 (0.00)	
Grade 4	0 (0.00)	0 (0.00)	
Grade 5	1 (0.49)	0 (0.00)	
Conversion	10 (4.90)	0 (0.00)	0.088
Total	39 (19.12)	5 (8.77)	*0.042*

Categories based on the Clavien-Dindo classification [17]

Level of significance *p* < 0.05 rendered in italics

*Chi^2^ test

## Discussion

In this retrospective multicenter study, the outcome of PRA was compared with that of LTA. PRA turned out to be superior to LTA in terms of operation time and independent of tumor size. Furthermore, complication rates were lower following PRA. Our findings appear to encourage the utilization of the PRA in patients with small- to medium-sized adrenal tumors; the procedure also seemed feasible for large (>8 cm) tumors.

Previous studies have shown varying results. In several studies, no significant differences in outcome variables were noted between the laparoscopic PRA versus the “classic” laparoscopic LTA [5, 19–21]. Similar operation times were reported in early studies. However, more recent studies reported shorter surgery duration for PRA than for LTA [22–24]. Moreover, less intraoperative blood loss had already been described for PRA [22], as well as lower postoperative pain [25], and shorter hospital stay [22–25]. These findings are consistent with our results, except for recovery time which did not differ between the two different approaches. One of the probable explanations is that most patients were surgically and physically fit enough to be discharged but demanded longer biochemical monitoring by the endocrinologist to assess adrenal function and to prevent hypoaldosteronism. Because we have looked at the total admission days, we could have missed a potential difference in recovery time.

The retroperitoneoscopy was first introduced in 1969 by Bartel [26]; however, at that time, the endoscopic instrumentation was of far less quality then it is now. In 1995, Walz et al. [27] reintroduced the retroperitoneoscopy for adrenalectomy. After developing and standardizing the procedure, it gained popularity worldwide and will probably become the procedure of choice for small- to medium-sized benign adrenal tumors in the future [14, 22, 25, 28, 29]. For large benign adrenal tumors, PRA seemed difficult because of the limited retroperitoneal space. However, in this study, we found a shorter operation time in PRA compared to LTA independent of tumor size, also for large (>8 cm) tumors. PRA seemed safe and feasible for selected large (>8 cm) benign tumors.

The location of the adrenal glands in the retroperitoneal space and the direct approach explains the shorter operation times and lower amount of blood loss in PRA compared to LTA. In the PRA, the adrenal is approached directly in the retroperitoneal space, without the need to mobilize and dissect adjacent intra-abdominal organs such as the liver, pancreatic tail, or spleen. Simply stated, one can do no harm to an organ if it is not encountered or mobilized during the procedure, which is also reflected by complication rate differences. Other advantages of the retroperitoneal approach are the avoidance of peritoneal adhesions in patients with a previous history of abdominal surgery, easier bilateral adrenalectomy in one surgical session without the need for repositioning, and lastly, its feasibility in obese patients as the abdominal fat is located at the non-operative ventral side of the patient. However, most surgeons are not familiar with the anatomic environment of the retroperitoneal space, and therefore, training in the use of PRA requires a substantial learning time [30]. For an experienced endoscopic surgeon, the learning curve will mostly be due to reorientation in a new anatomical environment: the inflated retroperitoneal space. A comprehensive training can shorten the learning period; about 20–25 PRAs would probably be necessary to apprehend the new technique [28]. Walz et al. [14] showed that the retroperitoneal approach is difficult to perform in patients with large tumors (>7–8 cm) and in patients with a high BMI. This is contrary to the present findings, which show no correlation between BMI and the outcome measures (operation time, recovery time, and blood loss). Also in the UMCU, the PRA procedure seemed safe for large tumors. Furthermore, concerns have been raised regarding the higher CO2 pressure needed for PRA (20–25 mmHg). However, neither in this nor in previous studies, CO2 related complications, such as CO2 embolism, were described for PRA [14, 22].

Although this study confirmed the superiority of the PRA, it does have several limitations that need to be addressed. First of all, the fact that our study has a retrospective nature; our data may be biased by variations in the recording methods used in the electronic patient record systems. Not all patient factors (preoperative performance status) that could influence the outcome measures were available for the analyses. Moreover, just like most retrospective studies, there are missing data, and secondly, there is a selection bias in time as patients treated by LTA ranged from 1998 to 2013 whilst patients treated by PRA ranged from 2008 to 2013. Since 1998, major improvements have been made by the introduction of advanced endoscopic devices like the Ligasure, Ultracision, and the Thunderbeat, as well as the Nathanson retractor. These developments could have influenced operation time. Despite this, we did not find a significant difference in operation time, recovery time, and complications in LTA between 1998 and 2007 compared to 2008–2013. Furthermore, operation time in PRA was significantly shorter than operation time in LTA in patients treated between 2008 and 2013, independent of tumor size. Lastly, the study did not take the varying experience of different surgeons into account. In the UMCU, mostly one surgeon operated, whilst in the UMCG, more than 93% of operations were performed or supervised by the same experienced surgeon who initiated the laparoscopic program. Subgroup analysis showed no significant difference in outcome parameters between operations performed by the main experienced surgeon and operations performed in a teaching setting.

Following the results of the present data, the UMCG recently started to implement the posterior retroperitoneal approach. Not only the reorientation in the retroperitoneal anatomy but also the unfamiliarity with the patient positioning and set-up requires a learning curve and an adaptation to this new technique. The ideal approach for implementing and apprehending a new technique involves on-site supervision of a surgeon–mentor or proctor. However, this is not always feasible. Remote telementoring could be a safe and feasible alternative to assist surgeons in safely introducing a new technique [31].

## Conclusion

Application of the PRA decreases operation times and blood loss compared to LTA and associated complication rates are significantly lower for PRA (19.1 versus 8.8% for LTA). This might encourage institutions that currently use the LTA to start using PRA in patients with adrenal tumors, independent of tumor size.

### Author’s contributions

- Study conception and design: Vrielink, Kist, and de Vries. - Acquisition of data: Vrielink, Kist, Borel Rinkes, Vries, and Kruijff. - Analysis and interpretation of data: Vrielink, Wevers, and Kruijff. - Drafting of manuscript: Vrielink, Wevers, Vriens, and de Vries. - Critical revision of manuscript: Vrielink, Wevers, Kist, Borel Rinkes, Hemmer, Vriens, de Vries, and Kruijff.
